# The Research Progress of Antiangiogenic Therapy, Immune Therapy and Tumor Microenvironment

**DOI:** 10.3389/fimmu.2022.802846

**Published:** 2022-02-23

**Authors:** Haoyue Hu, Yue Chen, Songtao Tan, Silin Wu, Yan Huang, Shengya Fu, Feng Luo, Jun He

**Affiliations:** ^1^Lung Cancer Center, Cancer Center, State Key Laboratory of Biotherapy, West China Hospital of Sichuan University, Chengdu, China; ^2^Department of Medical Oncology, Sichuan Cancer Hospital and Institute, Sichuan Cancer Center, Medicine School of University of Electronic Science and Technology, Chengdu, China; ^3^Department of Pathology, Beijing Shijitan Hospital, Capital Medical University, Beijing, China; ^4^School of Pharmacy, Southwest Medical University, Luzhou, China; ^5^Second Department of Oncology, Sichuan Friendship Hospital, Chengdu, China; ^6^Department of Oncology, The Third Hospital of Mianyang (Sichuan Mental Health Center), Mianyang, China

**Keywords:** antiangiogenic therapy, immune therapy, tumor microenvironment, cancer biology, progress

## Abstract

Anti-angiogenesis therapy, a promising strategy against cancer progression, is limited by drug-resistance, which could be attributed to changes within the tumor microenvironment. Studies have increasingly shown that combining anti-angiogenesis drugs with immunotherapy synergistically inhibits tumor growth and progression. Combination of anti-angiogenesis therapy and immunotherapy are well-established therapeutic options among solid tumors, such as non-small cell lung cancer, hepatic cell carcinoma, and renal cell carcinoma. However, this combination has achieved an unsatisfactory effect among some tumors, such as breast cancer, glioblastoma, and pancreatic ductal adenocarcinoma. Therefore, resistance to anti-angiogenesis agents, as well as a lack of biomarkers, remains a challenge. In this review, the current anti-angiogenesis therapies and corresponding drug-resistance, the relationship between tumor microenvironment and immunotherapy, and the latest progress on the combination of both therapeutic modalities are discussed. The aim of this review is to discuss whether the combination of anti-angiogenesis therapy and immunotherapy can exert synergistic antitumor effects, which can provide a basis to exploring new targets and developing more advanced strategies.

## Current Status of Anti-Tumor Angiogenesis Therapy

**Figure 1:**
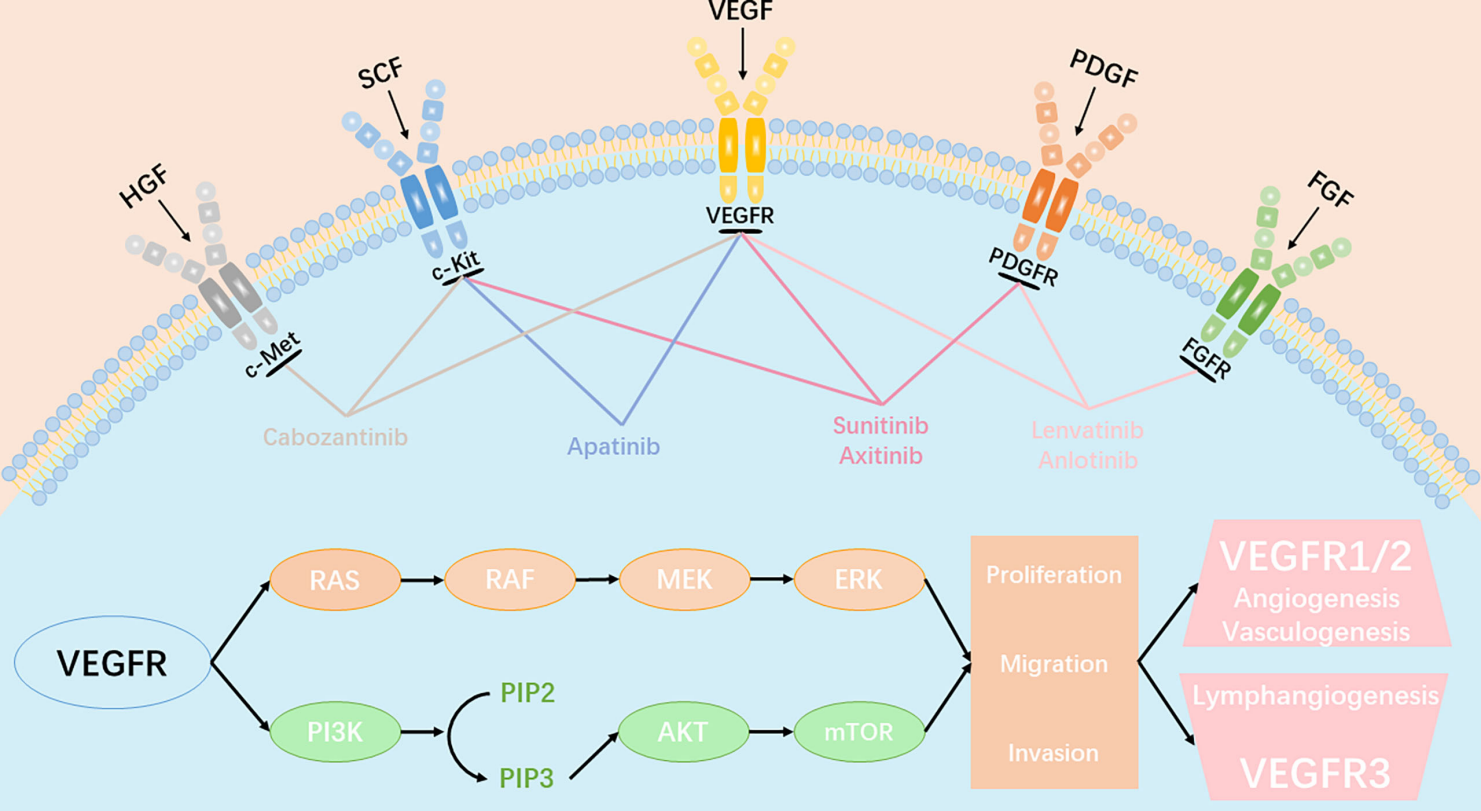
Angiogenic signaling pathway and key anti-angiogenic targets in tumor angiogenesis. VEGFR、PDGFR、FGFR、c-kit、c-Met involved in the key molecular signal events(RAS-RAF-MEK-ERK signaling pathway and PI3K-AKT-mTOR signaling pathway) which plays a significant role in tumor proliferation, migration and invasion. All approved angiogenic tyrosine kinase inhibitors (TKIs) can target multiple receptors simultaneously and inhibit the transduction of downstream signaling.

In 1971, Folkman hypothesized that “neovascularization is critical for tumors growth”. Since then, anti-tumor angiogenesis therapy has gained considerable attention, and is currently one of the most effective methods to treat cancer. Tumor blood vessels are fundamental for tumor growth and metastasis. Tumor angiogenesis is regulated by a variety of cytokines. The vascular endothelial growth factor (VEGF) family regulates the growth of blood vessels. In mammals, there are five isoforms within the VEGF family, including VEGF-A, VEGF-B, VEGF-C, VEGF-D, and placental growth factor (PGF). These proteins correspond to three different tyrosine kinase receptors, known as VEGFR1, VEGFR2, and VEGFR3. Studies have demonstrated that VEGF is highly expressed in different types of tumors, including vascular endothelial growth factor receptor (VEGFR1), VEGFR2, and VEGFR3. VEGFR2 plays a significant role in angiogenesis. VEGFR2 can activate the MAPK and PI3K signal pathways, which, in turn, activates the downstream ERK1/2 or mTOR ligand, leading to tumor growth and angiogenesis (**Figure 1**). Therefore, most anti-angiogenic drugs target the VEGF signaling system (ligands, receptors, and intracellular downstream pathways). Over the past 20 years, dozens of antiangiogenic drugs have been granted approval for treatment of multiple cancer types. One meta-analysis of randomized phase II/III trials (1) showed that, compared to platinum-based chemotherapy alone, the combination of bevacizumab and chemotherapy significantly prolonged survival of previously untreated patients with advanced non-small-cell lung cancer (NSCLC) (HR = 0.90, 95% CI: 0. 81-0. 99, *P* = 20). In the phase III LUME - Lung1 trial, the combination of docetaxel and nintedanib increased the median overall survival (OS) of lung adenocarcinoma patients, who had relapsed within 9 months of first-line chemotherapy, from 7.9 months to 10. 9 months (HR = 0.75, 95% CI: 0.60- 0.92, *P* = 0. 0073) (2). In the second cohort, the combination treatment also provided a survival benefit (median OS of docetaxel + nintedanib group *vs* control group = 12.6 months *vs* 10.3 months; HR = 0.83, CI:0.70-0.99, *P* = 0. 0359). The phase III REVEL trial (3) compared the therapeutic effect of docetaxel alone, as well as in combination with ramucirumab, in advanced NSCLC patients recalcitrant to platinum-based dual-drug chemotherapy. The median survival duration of the combination treatment group was 10.5 months, while the docetaxel group was 9.9 months (HR = 0.86, 95% CI: 0.75-0.98, *P* = 0.023). Subsequently, docetaxel and ramucirumab were granted approval by both EMA and FDA for treating metastatic NSCLC.

However, anti-tumor angiogenesis therapy has shown limited efficacy, with survival benefits ranging from only a few weeks to months. On the other hand, other studies have reported tumor progression (4) during anti-angiogenesis treatment. For example, Kindler et al. (5) compared the therapeutic effect of gemcitabine alone and combined with bevacizumab on advanced pancreatic cancer patients, and found that the combination therapy did not improve OS. Similarly, a significant recurrence rate was seen among glioma patients treated with bevacizumab (6). Studies on xenograft models of melanoma or breast cancer (7) showed that sunitinib, in fact, increased metastasis and shortened the survival of the tumor-bearing mice. Recently, the China Food and Drug Administration (CFDA) approved the single agent anlotinib as a third-line treatment for patients with advanced NSCLC. Compared to placebo, anlotinib has been demonstrated to improve both progression-free survival (PFS) and overall survival (OS) in a phase III trial among patients with advanced non-small-cell lung cancer (NSCLC), despite progression of cancer after two lines of prior treatments.

## The Resistance to Antiangiogenic Therapy

Among normal healthy human beings, angiogenic balance exists due to regulation of the vascular endothelial growth factors (VEGFs) - angiostatin and the angiogenic molecules. However, in tumors, this balance becomes converted to pro-angiogenesis. VEGFs, which play pivotal roles in wound healing and angiogenesis, consist of five members, VEGF A-E. VEGF works by binding to the vascular endothelial growth factor receptor (VEGFR), which is comprised of three tyrosine kinases. VEGA-A is up-regulated in most solid tumors, including breast cancer and lung cancer, which makes it a significant target of anti-angiogenesis drugs (8). The first FDA-approved anti-angiogenesis treatment was kinase inhibitors that targeted the VEGFR. Subsequently, antibodies against VEGF-Trap and VEGFR2 have been approved (9). Hepatocyte growth factor (HGF) promotes cell survival, enhances cell invasion ability, and facilitates epithelial-mesenchymal transition by activating the mesenchymal-epithelial transformation factor (c-MET) signaling pathway in endothelial cells. To date, researchers have discovered that c-MET and HGF are overexpressed in a number of tumors, which has led to abnormal gene amplification, activation of transcription or hypoxia microenvironment. Aberrant HGF/Met signaling is associated with poor prognosis in several tumor types. HGF/Met signaling stimulates several pathways, including MAPK signal pathway, which we mentioned above. PI3K signal pathway and Wnt/β-catenin pathways play significant roles in cell proliferation, survival, and angiogenesis. However, clinical efficacy of VEGF-targeted drugs has vital limitations. Although phase 3 trials have demonstrated that use of anti-angiogenic agents leads to significant improvements in overall survival (OS) for several cancers, such as advanced-stage CRC, RCC, and HCC, it is also associated with a failure to improve OS in other cancers, such as breast cancer, glioblastoma, pancreatic ductal adenocarcinoma (PDAC) and prostate cancer (10–13). Carvalho B et al. discovered that, in glioblastoma, c-Met overexpression is associated with a time-to-progression (TTP) after bevacizumab of 3 months (95% CI, 1.5–4.5), compared with a TTP of 7 months (95% CI, 4.6–9.4) among patients with low or no expression of c-Met (*p* = 0.05). VEGFR2 expression was associated with a TTP after bevacizumab treatment at 3 months (95% CI, 1.8–4.2) compared with a TTP at 7 months (95% CI, 5.7–8.3) among patients with no expression of VEGFR2 (*p* = 0.009). Concomitant c-Met/VEGFR2 overexpression was found to be associated with worse overall survival (13 months) compared to concomitant c-Met/VEGFR2 negative expression (19 months; *p* = 0.025). Their data indicates that c-Met and VEGFR2 overexpression play a significant role in the development of glioblastoma early resistance and may predict poorer responses to anti-angiogenic therapies (13).

Either anti-VEGFs or anti-VEGFRs or other nonspecific tyrosine kinase inhibitors ultimately shut down tumor blood supply and drive tumor necrosis. Necrosis usually occurs in the central part of the tumor, and the surrounding tumor cells remain alive as they are benefited by nutrition delivered by nearby normal blood vessels. As a result, most vascular disruptor therapy does not completely prevent tumor growth. This may be one of the reasons why antiangiogenic therapy improves therapeutic outcomes, while beneficial effects remain short (9). In addition, primary or acquired resistance contributes more to failure of anti-angiogenesis treatment. Tina Cascone used mouse-and human-specific profiling of human NSCLC xenografts in mice in order to investigate stromal and tumor cell changes that occur in tumors that acquire resistance to anti-angiogenesis treatment. Researchers found that changes in gene expression, particularly changes in expression of angiogenesis-related genes, occurred predominantly in stromal cells, but not in tumor cells. The observation reinforces the notion that tumor stroma may play an important and potentially dominant role, in at least some circumstances — in VEGF inhibitor resistance (14). Furthermore, extrinsic mechanisms have also been shown to be involved in resistance to antiangiogenic therapy, including changes in the tumor microenvironment (TME), the presence of cancer stem cells (CSCs), and tumor immunosuppression, which significantly limits their clinical value (15, 16).

## Tumor Microenvironment and Antiangiogenic Therapy

**Figure 2:**
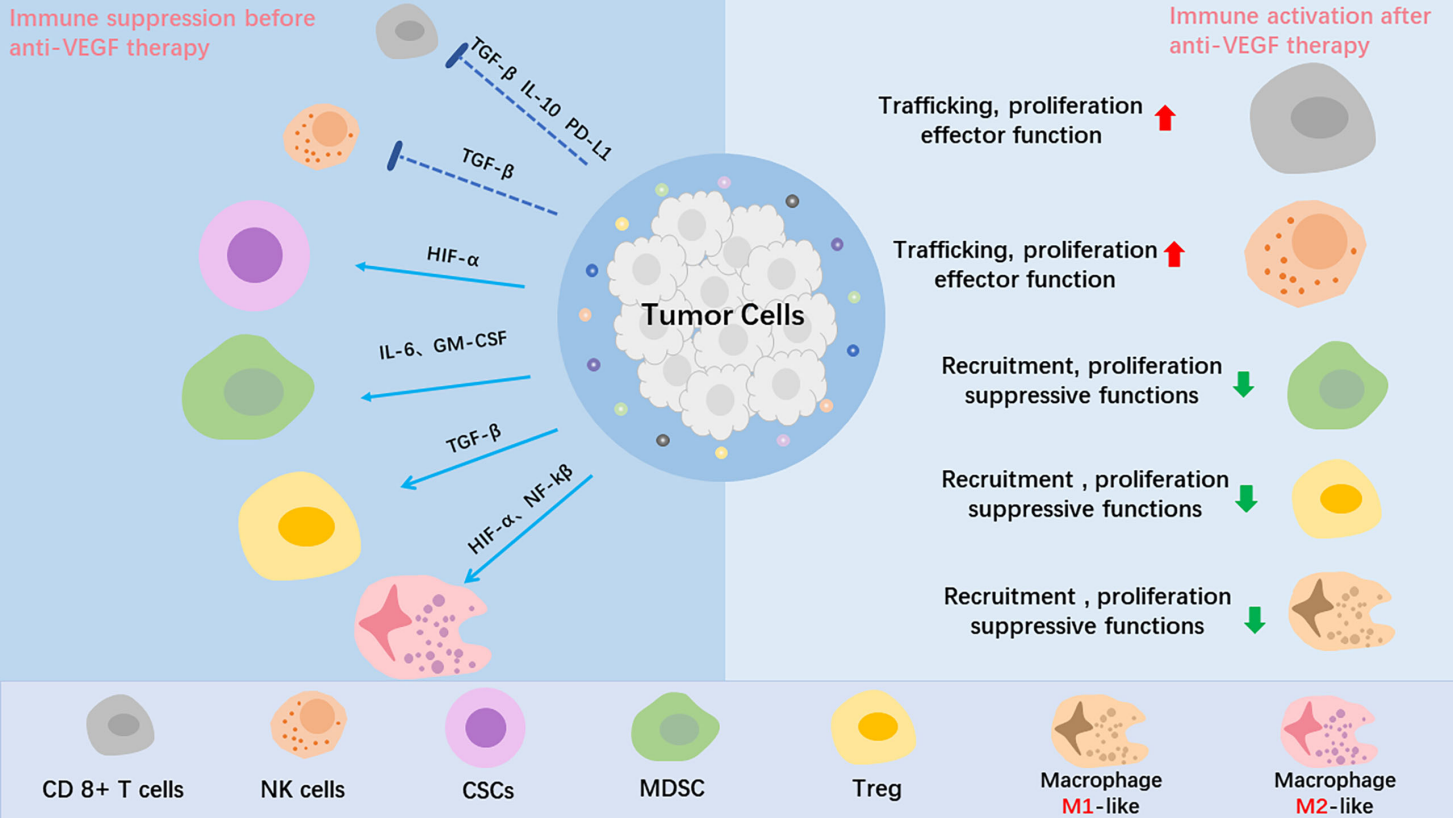
The role of anti-VEGF treatment in the tumor microenvironment (TME). Tumor angiogenesis creates a hypoxic tumor microenvironment, which impedes T-effector cells、NK cells and DC cells infiltration into tumor, mediates tumor cell de-differentiation into CSCs, promotes proliferation of immunosuppressive cells, including Tregs and MDSCs, and polarizes TAMs to the immune inhibitory M2-like phenotype. After anti-VEGF treatment, the anti-tumor factors increase, and the pro-tumor factors are decreased. In summary, anti-VEGF treatment alleviate the immunosuppressive tumor microenvironment and improve cancer immunotherapy.

The tumor microenvironment (TME) is composed of immune cells, stromal cells, extracellular matrix (ECM), blood vessels, tumor cells, lymphatic vessels and CSCs. The constant changes that occur in the various components of the TME result in its complexity and heterogeneity. TME is associated with multiple processes, including proliferation, angiogenesis, apoptosis, and immune surveillance. The stromal cells, particularly cancer-associated fibroblasts (CAFs), can promote tumor cell survival mainly by recruiting immune cells into the TME, and promote invasion by constructing a hypoxic environment. Tumor-driven hypoxia, increased inflammation, or MMPs overexpression in the TME induces alterations in the ECM, following the tumor biological behavior of evading apoptosis, elevating invasion and metastasis (17, 18). In addition, ECM components can regulate the cancer-immunity cycle. The above TME changes cause tumor progression and drug resistance (19). As tumors generally tend to be hypoxic, prolonged use of anti-angiogenesis drugs can often aggravate hypoxia (20, 21). As previously reported, the upregulation of hypoxic inducible factor 1a (HIF-1a) is also responsible for heterogeneity of breast cancer, lung cancer, cervical carcinoma, and gliomas (22–24). Hypoxia-induced upregulation of HIF-1a can mediate tumor cell de-differentiation into CSCs, which is a primary mechanism that underlies resistance to anti-angiogenesis therapy (25, 26). In addition, HIF-1a upregulates the expression of nuclear factor-kβ and leads to increased recruitment of monocytes and tumor-associated macrophages (TAMs), including polarization of the M2 phenotype TAMs, which promotes recurrence and metastasis (27). The immune system can have a dual effect in cancer biology, including pro-tumorigenic and anti-tumorigenic effect. The immune surveillance system identifies, kills, and removes tumor cells from the body. NK cells, CD8+ cytotoxic T cells and the major histocompatibility complex (MHC) class 1 molecules are known to play major roles in the function of immunosurveillance. Unsurprisingly, the host immune system is often disrupted and creates an immune imbalance among cancer patients. Tumor cells can camouflage themselves in order to hide from immune cells, thus avoiding being discovered. Numerous cellular and molecular mechanisms have been shown to be responsible for tumor evasion (28, 29). Immunosuppressive cells, such as T-regs, TAMs, and MDSCs frequently accumulate within the TME, which is associated with an unfavorable prognosis. When there are a large number of immune cells in tumor tissues, such as T-regs, MDSCs, TAMs, and DCs, they can promote an immunosuppressive microenvironment and participate in immune escape. Anti-angiogenic therapy for VEGF or VEGF receptor-2 (VEGFR-2) can increase the transport of T cells to the tumor, thereby reducing immunosuppressive cytokines and T-regs, helping overcome resistance to the checkpoint inhibitors’ medicinal properties (30) (**Figure 2**). Furthermore, tumor cells also secrete immunosuppressive cytokines, such as IL-10 and TGF-β, which are known to inhibit perforin and production by CD8+ T cells and inactivate cytotoxicity of NK cells (31–33).

Additionally, primary drug resistance due to a lack of tumor-infiltrating lymphocytes in the tumor should not be ignored. In addition to the VEGF pathway, angiogenesis can be induced by the angiopoietin (Ang1-2)/Tie-2 pathway. Studies have shown that patients that receive immunotherapy with higher Ang-2 expression tend to have poorer clinical outcomes. This suggests that the Ang-2 pathway is another cause of immunotherapy resistance (34, 35).

## The Efficacy of Anti-Cancer Immunotherapy

Under normal circumstances, the immune system can recognize and eliminate tumor cells within the tumor microenvironment. However, in order to survive, tumor cells can adopt different strategies in order to suppress the immune system and protect itself from CD8+ T cells. Immunotherapy has heralded a new era of oncotherapy and aims to either directly eliminate cancer cells or activate the host immune response. It is mediated through anti-cancer cell vaccines and antibodies, cytokines, adoptive immune cell transfer and immune checkpoint blockers (ICBs). Tumor immunotherapy is a treatment that restores the body’s normal anti-tumor immune response by restarting and maintaining the tumor-immune cycle, thereby controlling and eliminating tumor cells. Tumor immunotherapy includes monoclonal antibody immune checkpoint inhibitors (36), therapeutic antibodies, cancer vaccines, cell therapy and small molecule inhibitors. In recent years, cancer immunotherapy has continued to progress. At present, this treatment method has shown strong anti-tumor activity in the treatment of solid tumors such as melanoma, NSCLC, kidney cancer, and prostate cancer. Furthermore, immunotherapy drugs have been approved by the US FDA (Food and Drug Administration) for clinical application (37). Moreover, increasing evidence has shown that overexpression of vascular growth factors can activate immunosuppressive cells directly and suppress immune effector cells to alter the immunosuppressive microenvironment.

## Combination of Immunotherapy and Anti-Tumor Angiogenesis

The relationship between angiogenesis and immune therapy is a complicated interplay. Anti-angiogenic agents can stimulate the immune system and improve the immunosuppressive environment, while immunotherapy can also have anti-angiogenesis effects. Therefore, there is a synergistic relationship between the two treatment methods (38, 39). Tumor cells can evade T cell-mediated killing by up-regulating the interaction of PD-L1 with the inhibitory receptor PD-1, which is expressed on tumor-infiltrating T-cells. Tumor cells can evade T cell-mediated killing by upregulating the interaction of ligands (such as PD-L1) with the inhibitory receptor PD-1, CTLA-4, and LAG-3, which are expressed on tumor-infiltrating T-cells (40). It is inevitable that patients will develop resistance to immune checkpoint inhibitors due to a lack of PD-L1 and the inhibitory effect in the TME. Facing a complex TME, the key strategy is to inhibit angiogenesis, and an effective immune response (41).

### Anti-Angiogenesis Produces Vessel Normalization and Stimulates Immune Responses

The formation of blood vessels in malignant tumors is largely caused by hypoxia and the excessive secretion of VEGF. Anti-angiogenic therapy for VEGF or VEGFR-2 can increase the transport of T cells to the tumor, thereby reducing immunosuppressive cytokines and regulatory T cells, which may help overcome resistance to checkpoint inhibitors’ medicinal properties (30). A case study of immune checkpoint inhibitors combined with anti-angiogenic drugs in the treatment of metastatic renal cell carcinoma demonstrated that antigen-specific T cell migration and expression of MHC-1 and PD-L1 were increased. Furthermore, anti-tumor activity was enhanced with less toxicity (42). Tumor blood vessels were found to be highly abnormal, with tumor vessels showing structural abnormalities, leading to hypoxia, acidity, and a high interstitial fluid pressure microenvironment. These microenvironmental abnormalities can affect immune cell proliferation, infiltration, survival, and function (43). Myeloid-derived suppressor cells (MDSCs) are one of the most important stromal cells of the TME, and protect tumor cells from the host immune system by suppressing T-cell function (44). There is evidence to support the hypothesis that anti-angiogenic therapy and immunotherapy act synergistically (45). GM-CSF, a potent cytokine promoting the differentiation of myeloid cells such as dendritic cells, macrophages and granulocytes, which elicits antitumor immunity by enhance tumor antigen presentation to T cells, has been proven to be effective across numerous clinical trials (46–50).

Another study has reported that binding of tumor-derived VEGF to VEGFR on CD34+ bone marrow progenitor cells reduces the differentiation of these cells into dendritic cells, thus limiting the efficacy of GM-CSF-related immunotherapy (51). And Sylvie et al. also included that human GFs *in vitro* actively inhibit the differentiation of monocyte-derived dendritic cells through the secretion of IL-6 and VEGF, limiting the immunotherapy of GM-CSF (52). Furthermore, it also promotes proliferation of immunosuppressive cells, such as Tregs and MDSCs, and inhibits DC maturation, and restricts the development of T lymphocytes from the lymphoid progenitors (30, 53–55). A study (56) on three different NSCLC animal models demonstrated that combining adoptive transfer of cytokine-induced killer (CIK) cells with recombinant human endostatin significantly inhibited angiogenesis and tumor growth, whereas neither was effective when used alone.

### Blocking VEGF Induced Immune Checkpoint Expression

Lydia Meder et al. conducted an experiment on five groups on the combined use of vehicle, IgG, VEGF inhibitor, PD-L1 inhibitor, VEGF inhibitor, and PD-L1 inhibitor in a mouse model of small cell lung cancer. The results indicate that treatment with VEGF, compared to any other treatment methods, the combination of inhibitor and PD-L1 inhibitor greatly improved PFS and OS in mice (57). S. Yasuda et al. reported that in a mouse model of colon cancer, the combined use of PD-1 inhibitors and VEGFR2 inhibitors demonstrated no obvious toxicity. Compared to the control group, the experimental group drugs were found to better inhibit tumor growth. The author believes that the combined use of inhibitors can produce a synergistic anti-tumor effect in the body through a variety of mechanisms, including anti-VEGFR2 therapy resulted in a significant decrease of tumor micro vessels as well as reducing tumor vasculature and anti-PD-1 mAb treatment enhanced the infiltration of T cells into tumors. And that the two drugs are not mutually exclusive (58). Since immunotherapy has been proved to be effective against CSCs and the immunosuppressive TME, it is reasonable to surmise that a combination of anti-angiogenesis and immunotherapies would have a synergistic effect against recalcitrant tumors. Indeed, studies have shown that (38) targeting the angiogenic factor VEGF, as well as its receptors, stimulates onco-immunity, since VEGF is known to be involved in the immune escape of tumors. The VEGF signaling pathway can abrogate the effects of anti-tumor therapy *via* various mechanisms. Usually, is LFA1 that can interact on ICAM1. LFA1 is expressed on lymphocytes and it is a crucial for T cell entry into mammalian lymph nodes and tissues while ICAM1 on tumor target cells or endothelial cells (59). Previous study showed that clustering of ICAM-1 was indeed prevented by VEGF and a reduced induction of ICAM-1 and VCAM-1 mRNA transcripts by TNF in the presence of VEGF (60). VEGF can also block T-cell activation and induce apoptosis *via* the Fas/FasL pathway (61). Therefore, blocking VEGF and its receptor can help stimulate immune responses and improve immunotherapy outcomes. Studies on tumor-bearing mouse models have demonstrated that (62, 63) multi-targeted anti-angiogenic tyrosine kinase inhibitors (TKIs) increased tumor infiltration of CD8+ and CD4+ T cells by downregulating PD-1 expression, and decreased the number and activity of Tregs and MDSCs (64). Similarly, sunitinib inhibited the expansion of Tregs and MDSCs in patients with renal cell carcinoma (30, 63, 65). The VEGFR2-targeting TKI cabozantinib was also associated with a reduction in the number of Tregs and MDSCs, and simultaneously promoted tumor infiltration of CD4+ and CD8+ T lymphocytes, both alone and in combination with the anti-cancer vaccine MVA/rF-CEA/TRICOM (65). VEGFR1/R2 and soluble chimeric VEGF receptor, can bind to VEGF with high affinity and efficiently play an anti-angiogenic therapy function. In animal experiments, the combination of sVEGFR1/R2 therapy and GM-CSF–secreting tumor cell immunotherapy can remarkably prolong the survival of tumor model mice (66, 67). Similarly, the combination of cabozantinib and MVA/rF-CEA/TRICOM was found to significantly inhibit growth of MC38-CEA tumors in a mouse model (65). In a mouse model of colon cancer, anti-PD-1 monoclonal antibodies and VEGFR2 resulted in significantly greater tumor inhibition compared to either monotherapy (58).

## Clinical Use of Antiangiogenic Drugs and ICBs Against Tumors

The relationship between angiogenesis and immune therapy has been suggested to be a complicated interplay (68). Anti-angiogenic agents are known to stimulate the immune system and improve the immune suppression environment (69). Furthermore, immunotherapy can also cause anti-angiogenesis effects, and there is a synergistic relationship between the two treatment methods (39). Tumor cells can evade T cell-mediated killing by up-regulating the interaction of PD-L1 with the inhibitory receptor PD-1 that is expressed on tumor-infiltrating T cells. Thus, it is inevitable that patients develop resistance to immune checkpoint inhibitors due to a lack of PD-L1 and the inhibitory effect in the tumor microenvironment. The therapy should inhibit angiogenesis, on the other hand trigger anti-tumor immunity (41). The formation of blood vessels in malignant tumors is mainly caused by hypoxia and excessive secretion of vascular endothelial growth factor (VEGF). Recently, an accumulating number of clinical trials have been conducted to explore the efficacy of the combination of anti-angiogenesis and immunotherapy (**Table 1**).

**Table 1:** Principal clinical trials for the approval of antiangiogenic and or immunotherapy agents.

Drug	Indication	Phase	Pivotal study	PFS (Months)	OS (Months)	ORR	First posted	Recruitment status
Atezolizumab + Bevacizumab	NSCLC	3	NCT02366143	8.3 vs 6.8	19.2 vs 14.7	NA	2015	Completed
Bevacizumab + Nivolumab	NSCLC	1	NCT01454102	9.3 vs 4.0	21.7 vs 14.1	8.0% vs 10.0%	2011	Completed
Ramucirumab + Pembrolizumab	NSCLC	1	NCT02443324	9.7	26.2	30.0%	2015	Active, not recruiting
Ramucirumab + Durvalumab	NSCLC	1	NCT02572687	2.7	11.0	11.0%	2015	Completed
Bevacizumab + Atezolizumab	RCC	2	NCT01984242	11.7 vs 8.4 vs 6.1	NA	32.0% vs 29.0% vs 25.0%	2013	Completed
Nivolumab + Ipilimumab	RCC	3	NCT02231749	11.6 vs 8.4	NA vs 26.0	9.0% vs 1.0%	2014	Active, not recruiting
Axitinib + Avelumab	RCC	1	NCT02493751	NA	NA	27.0% vs 4.0%	2015	Completed
Axitinib + Avelumab	RCC	3	NCT02684006	13.8 vs 8.4	11.6 vs 10.7	55.2% vs 25.5%	2016	Active, not recruiting
Pembrolizumab + Axitinib	RCC	3	NCT02853331	17.1 vs. 11.1	NA	60.0% vs. 38.5%	2016	Active, not recruiting
Tivozanib + Nivolumab	RCC	1/2	NCT03136627	18.9%	NA	56.0%	2017	Completed
Apatinib + SHR-1210	HCC	1	NCT02942329	2.9	11.4	30.8%	2016	Unknown
Bevacizumab + Atezolizumab	HCC	1	NCT02715531	5.6 vs 3.4	NA	36.0%	2016	Completed
Nivolumab + Ipilimumab	HCC	1/2	NCT01658878	NA	22.8 vs 12.5 vs 12.7	32.0% vs 27.0% vs 29.0%	2012	Active, not recruiting
Lenvatinib + Pembrolizumab	HCC	1	NCT03006926	8.6	22.0	36.0%	2016	Active, not recruiting
Bevacizumab + Atezolizumab	HCC	3	NCT03434379	NA	NA	NA	2018	Active, not recruiting
Bevacizumab + Dacarbazine	Melanoma	2	NCT01164007	5.5	11.4	18.9%	2010	Completed
Bevacizumab + Ipilimumab	Melanoma	1	NCT00790010	9.0	25.1	19.6%	2008	Active, not recruiting
Axitinib + Toripalimab	Melanoma	1	NCT03086174	7.5	NA	67.5%	2017	Active, not recruiting
Bevacizumab + Atezolizumab	CRC	2	NCT0287319	4.4 vs 3.3	NA	NA	Unknown	Unknown
Bevacizumab + Nivolumab	CRC	2	NCT04072198	NA	NA	NA	2019	Recruiting

NSCLC, non-small cell lung cancer; RCC, renal cell cancer; HCC, hepatocellular carcinoma; CRC, colorectal cancer; ORR, objective responses rate; RFS, progression-free survival; OS, overall survival. NA, Not available.

### Clinical Trials on NSCLC

In a phase 3 clinical trial IMpower150 (NCT02366143), 1202 patients with metastatic non-squamous NSCLC (ns-NSCLC) were treated with a combination of Atezolizumab to Bevacizumab-based chemotherapy, including three groups: (1) Atezolizumab, Carboplatin, and Paclitaxel (ACP); (2) Atezolizumab, Bevacizumab, Carboplatin and Paclitaxel (ABCP); (3) Bevacizumab, Carboplatin and Paclitaxel (BCP). The results demonstrated that the ABCP group had significantly improved PFS and OS, with an average of 8.3 and 19.2 months, respectively, both of which were better than the control group (70). Subgroup analysis was performed according to the status of EGFR, results of which indicated that the PFS of ABCP group (n=356) and BCP group (n=336) in EGFR wild-type (WT) population were 11.3 months and 6.8 months, respectively. Furthermore, 124/1202 EGFR-positive patients were randomized to three groups, including the ABCP (n=34), ACP (n=45), or BCP (n=45). The median OS was not estimated in the ABCP, but it was 21.4 months in the ACP group and 18.7 months in BCP group (71). In addition, patients with advanced NSCLC receiving treatment with a combination of Nivolumab and Bevacizumab were recruited for a phase 1 study (NCT01454102), which aimed to evaluate whether the combination therapy improves PFS and OS. The experimental results indicate that the combined treatment group had significant safety, and the incidence of grade 3 and above adverse reactions is low. Therefore, it has shown excellent therapeutic effects compared to the single-agent treatment group (72). The combined treatment group had a median PFS of 37.1 weeks; however, in the Nivolumab monotherapy group, the median PFS of squamous cell carcinoma was 16 weeks, and the median PFS of non-squamous cell carcinoma was 21.4 weeks. Additionally, the median OS of the combined treatment group was 86.7 weeks, which is much larger than that of the monotherapy group (73). In 2019, results of the phase 1 study (NCT02443324) were reported. Among the total 27 enrolled patients with previously treated advanced NSCLC that received Ramucirumab plus Pembrolizumab, 8 patients achieved an objective response. Results also demonstrated that the disease control rate was 86%, with a median PFS and OS of 9.7 and 26.2 months, respectively (74). Another phase 1 study (NCT02572687) indicated that the combination of Ramucirumab plus Durvalumab led to an enhancement of preliminary antitumor activity in heavy pre-treated NSCLC patients with a median PFS of 1.7 months and OS of 12.4 months (75).

### Clinical Trials on RCC

A first randomized phase 2 IMmotion150 study (NCT01984242) for patients with previously untreated mRCC treated with Atezolizumab combination Bevacizumab or single Atezolizumab or single sunitinib showed that the PFS of this combination group significantly improved within the population, whatever the PD-L1 status (76). Immunotherapy with PD-1 and PD-L1 inhibitors or combined with antiangiogenic therapy (i.e. VEGF inhibitors or CTLA-4 antibodies) has become a first line therapy for advanced RCC patients (77). Nivolumab plus Ipilimumab in a phase 3 trial (NCT02231749) has provided significant benefits in untreated intermediate and poor-risk RCC patients with a higher 18-month OS rate of 75% and objective responses rate (ORR) of 42%, compared to sunitinib with 18-month OS rate of 60% and ORR of 27% (78). In 2018, an open-label, dose-finding and dose-expansion multicenter phase 1 study (NCT02493751) confirmed the disease control rate (DCR) of 78% with the security in the combination therapy of axitinib plus Avelumab in advanced RCC patients (79). Another phase 3 study (NCT02684006) validated that the combination of Avelumab plus axitinib enhanced the curative effect in patients with advanced RCC, leading to remarkable improvement in median PFS (13.8 months) and ORR (51.4%), compared to treatment with sunitinib (8.4 months and 25.7%, respectively) (80). In 2020, a pivotal phase 3 study (NCT02853331) demonstrated that Avelumab or Pembrolizumab Plus axitinib were more efficacious than sunitinib, a previous standard of care. This study recruited 861 metastatic renal cell carcinoma (mRCC) patients with results showing an improvement in PFS, a high response rate, and a low rate of intrinsic resistance (81). Recently, a phase 1b/2 study (NCT03136627) of Tivozanib combined with Nivolumab in patients with mRCC has been completed. The results demonstrated a promising antitumor efficacy with ORR of 56%, DCR of 96% and median PFS of 18.9 months (82).

### Clinical Trials on HCC

Phase 1 study (NCT02942329) of the VEGFR2 inhibitor apatinib plus anti-PD1 antibody SHR-1210 in patients with advanced hepatocellular carcinoma (HCC) has demonstrated manageable toxicity and encouraged clinical activity at recommended single-agent doses of both drugs (83). A phase 1b clinical trial (NCT02715531) proved that combination of Bevacizumab and Atezolizumab profoundly improved the therapeutic effect compared to the standard-of-care sorafenib in a phase 3 trial with ORR and DCR of 62% and 78%, respectively. With regards to safety, the combination group demonstrated tolerable safety, with serious adverse events (AEs) rate of only 8% (84). CheckMate040 is a phase 1/2 randomized clinical trial (NCT01658878) which comprised of 148 HCC patients that were randomized 1:1:1 into three dosing arms (Nivolumab, alone or in combination with ipilimumab). These cohort results suggest that Nivolumab, plus Ipilimumab, may provide an improved ORR and OS, especially in arm A (lower dose Nivolumab and higher dose Ipilimumab), relative to anti-PD-L1 monotherapy (85). The combination of lenvatinib plus Pembrolizumab for unresectable HCC (uHCC) patients in the Phase 1b trial (NCT03006926) represented a promising antitumor activity with an ORR of 46.0%, and a median PFS of 9.3 months (86). Moreover, an ongoing double-blind randomized controlled phase 3 study (NCT03713593) of lenvatinib plus Pembrolizumab treatment of uHCC is currently being undertaken (77). Imbrave150 (NCT03434379), a randomized, multicenter phase 3 clinical study aims to evaluate the efficacy and safety of Atezolizumab plus Bevacizumab versus Sorafenib among patients with advanced HCC. The results indicated that, among 501 patients (336 in the combination group and 165 in the Sorafenib group) with HCC, the combination group showed a remarkable improvement in median PFS and OS with tolerated and controllable toxicity, compared to the Sorafenib group (87).

### Clinical Trials on Melanoma and CRC

Dacarbazine in combination with Bevacizumab was studied in a phase 2 study (NCT01164007) of 40 unresectable/metastatic melanoma patients. The results from this study indicated that the treatment had an ORR of 18.9% and a median OS of 11.4 months, with no new toxicity (88). Preliminary results from the phase 1 clinical trial (NCT00790010) showed that Ipilimumab (CTLA-4 antibody) plus Bevacizumab (VEGF inhibitors) in patients with metastatic melanoma (MM) had favorable clinical outcomes, for reasons of increasing tumor vascular expression of ICAM-1 and VCAM-1 and lymphocyte infiltration in tumors (89). Another open-label phase 1b trial (NCT03086174) validated the efficacy of axitinib in combination with Toripalimab among patients with advanced melanoma with an ORR of 48.3% and a median PFS of 7.5 months (90). In addition, a phase 2 study (NCT0287319) in 133 mCRC patients also demonstrated that the addition of Atezolizumab to Bevacizumab, as well as capecitabine, improved the median PFS of 4.4 months compared to that of 3.3 months in the modified intent-to-treat analysis (mITT) analysis group (91). Another open-label, multicenter phase 2 trial (NCT04072198) of FOLFOXIRI/Bevacizumab, in association with Nivolumab, was conducted in patients with mCRC. The results demonstrated that the combination was generally well tolerated, with an acceptable toxicity profile without any unexpected findings (92).

## Future Prospects

Anti-tumor angiogenesis was found to be favorable to T-cell infiltration and drug delivery to the tumor, thereby enhancing the efficacy of immunotherapy. Additionally, immunotherapy can also increase tumor vascular normalization and form positive feedback to anti-angiogenesis. Therefore, the combination of anti-angiogenic agents and immunotherapy provides a new therapeutic approach for tumor patients. A large number of studies have demonstrated that the combination therapy has good clinical application prospects. However, the relationship between tumor angiogenesis and immune response is intricate, and some tough problems still need to be solved for future practical application.

Firstly, there is no way to identify tumor patients that can benefit from combination therapy (93), and anti-angiogenesis therapy has a lack of biomarkers, as mentioned above. In order to address the problem, oncologists have to identify the biomarkers that can be associated with patient groups that would be advantaged with this therapy. Secondly, the dose of each drug, the optimal sequence, and the time of the combination also remain significant. The high or low dose, simultaneous or sequential treatment, will have an effect on the efficacy of the combination therapy. Furthermore, studies have demonstrated that high doses of anti-angiogenic drugs can directly damage tumor blood vessels, which results in more serious disturbances of tumor microenvironment, such as hypoxia and immunosuppression (94). Therefore, it is necessary to choose the appropriate drug dosage, and optimize the schedule of tumor immunotherapy and anti-angiogenesis therapy in order to obtain improved anticancer efficacy. Moreover, the most frequent side effect of anti-angiogenic is hypertension (95). Therefore, primary or acquired resistance, including non-upregulation VEGF in tumors, changes in the TME, the presence of CSCs, and the patient with hypertension contribute to anti-angiogenesis failure (16). Besides, resistance to immunotherapy, including lack of tumor-infiltrating lymphocytes in the tumor, accumulating immunosuppressive cells in the TME and secreting immunosuppressive cytokines in the tumor cells, contributes significantly to failure of immunotherapy.

## Author Contributions

FL and JH contributed to the study design. HH and YC were responsible for data collection. ST, YH, and SF drafted and prepared the manuscript. SW worked for the table and figures. All authors participated in the data interpretation and contributed to the manuscript writing with important intellectual input. All authors approved the final version of the manuscript.

## Conflict of Interest

The authors declare that the research was conducted in the absence of any commercial or financial relationships that could be construed as a potential conflict of interest.

## Publisher’s Note

All claims expressed in this article are solely those of the authors and do not necessarily represent those of their affiliated organizations, or those of the publisher, the editors and the reviewers. Any product that may be evaluated in this article, or claim that may be made by its manufacturer, is not guaranteed or endorsed by the publisher.
